# "The Terrible Ts" That Was Not: Anterior Mediastinal Tuberculosis Mimicking Malignancy in an Immunocompetent Young Adult

**DOI:** 10.7759/cureus.101776

**Published:** 2026-01-18

**Authors:** Atush Alipuria, Kamal Kishore Pandey, Ankur Gupta, Pooja Alipuria

**Affiliations:** 1 Respiratory Medicine, Yashoda Super Speciality Hospital, Ghaziabad, IND; 2 Internal Medicine, Yashoda Super Speciality Hospital, Ghaziabad, IND

**Keywords:** anterior mediastinal mass, antitubercular therapy, ct guided biopsy, extrapulmonary tuberculosis, immunocompetent host, mediastinal tuberculosis, necrotic mediastinal mass, tuberculosis mimicry

## Abstract

Anterior mediastinal masses are typically approached through the classic "terrible Ts" differential (thymic tumors, lymphoma, germ cell tumors, and thyroid disease). Tuberculosis (TB) rarely presents as an isolated anterior mediastinal mass in an immunocompetent adult and can mimic malignancy. We report the case of a male patient in his late 20s who presented with a two-month persistent dry cough, low-grade fever, and 8 kg of unintentional weight loss. Initial chest radiography demonstrated mediastinal widening, and high-resolution computed tomography (HRCT) revealed a large lobulated anterior mediastinal mass with central necrosis measuring 85 × 67 mm, abutting the pericardium and aortic arch, with subcarinal lymphadenopathy. Serum alpha-fetoprotein (AFP) and beta-human chorionic gonadotropin (β-hCG) were within normal limits (AFP: 3.59 ng/ml (reference range: 0.89-8.78); β-hCG: 1.50 mIU/ml (reference range: <5.00)). As imaging could not reliably distinguish thymic tumors, lymphoma, and necrotizing infectious disease, a CT-guided core biopsy was performed. Histopathology showed caseating granulomas with Ziehl-Neelsen-positive acid-fast bacilli, confirming the diagnosis of TB. The patient improved symptomatically within two weeks of antitubercular therapy (ATT). HRCT at two months showed regression to 70 × 62 × 35 mm. Serial chest radiographs showed continued improvement at six months and complete resolution at eight months, after which ATT was stopped. This case emphasizes that TB should remain in the differential diagnosis for necrotic anterior mediastinal masses in endemic settings and highlights the value of early tissue diagnosis to avoid unnecessary oncologic therapy or surgery.

## Introduction

Mediastinal masses encompass a wide spectrum of benign and malignant conditions, and accurate localization to a mediastinal compartment is central to diagnostic reasoning. The International Thymic Malignancy Interest Group (ITMIG) classification provides a widely adopted computed tomography (CT)-based framework that facilitates compartmental localization, narrows the differential diagnosis, and guides procedural planning [1]. In adults, anterior (prevascular) mediastinal masses account for a substantial proportion of mediastinal lesions and most commonly represent thymic epithelial tumors, lymphoma, or germ cell tumors, often prompting an initial malignancy-focused diagnostic approach. From an epidemiologic perspective, infectious etiologies constitute only a small minority of anterior mediastinal masses, particularly in immunocompetent individuals [1].

Nevertheless, in tuberculosis (TB)-endemic regions, extrapulmonary TB remains an important clinical mimic of malignancy and may present without pulmonary involvement. Isolated anterior mediastinal TB in immunocompetent patients is rare and has been primarily described through individual case reports, in which radiologic features frequently overlap with those of lymphoma or thymic neoplasms, creating significant diagnostic uncertainty [2,3]. Similar diagnostic challenges have been highlighted in prior reports describing young, immunocompetent patients with mediastinal TB initially suspected to be malignant [4]. Because therapeutic strategies differ substantially between infectious and malignant etiologies, relying solely on imaging findings may lead to delayed diagnosis or unnecessary invasive intervention.

We report the case of an immunocompetent young adult who presented with a large necrotic anterior mediastinal mass initially suggestive of malignancy. A definitive diagnosis was established through CT-guided core needle biopsy, and the patient achieved complete clinical and radiologic resolution following antitubercular therapy (ATT). This case underscores the importance of maintaining TB in the differential diagnosis of necrotic anterior mediastinal masses in endemic settings and highlights the critical role of early tissue confirmation in guiding appropriate management.

## Case presentation

A previously healthy male in his late 20s presented with a two-month history of persistent dry cough and unintentional weight loss of 8 kg, along with intermittent low-grade fever. He denied a history of hemoptysis, night sweats, known TB exposure, and immunosuppressive conditions. Physical examination showed tachycardia (108/min) and mild hypotension (100/90 mmHg) without respiratory distress. No peripheral lymphadenopathy was noted. Testicular and inguinal examinations revealed no palpable mass.

Investigations

**Figure 1:**
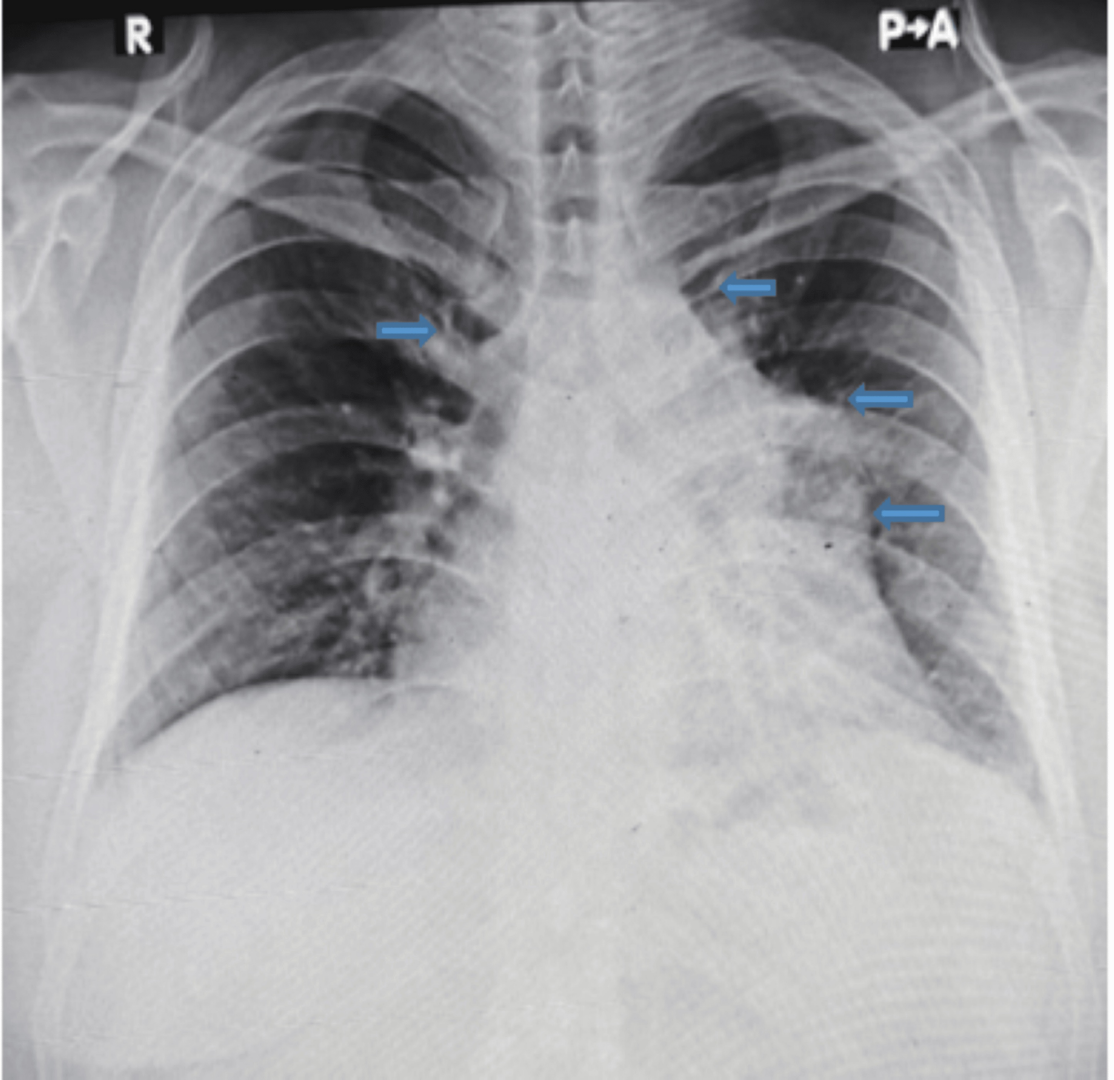
Chest radiograph (PA view) at presentation showing mediastinal widening PA, posteroanterior

**Figure 2:**
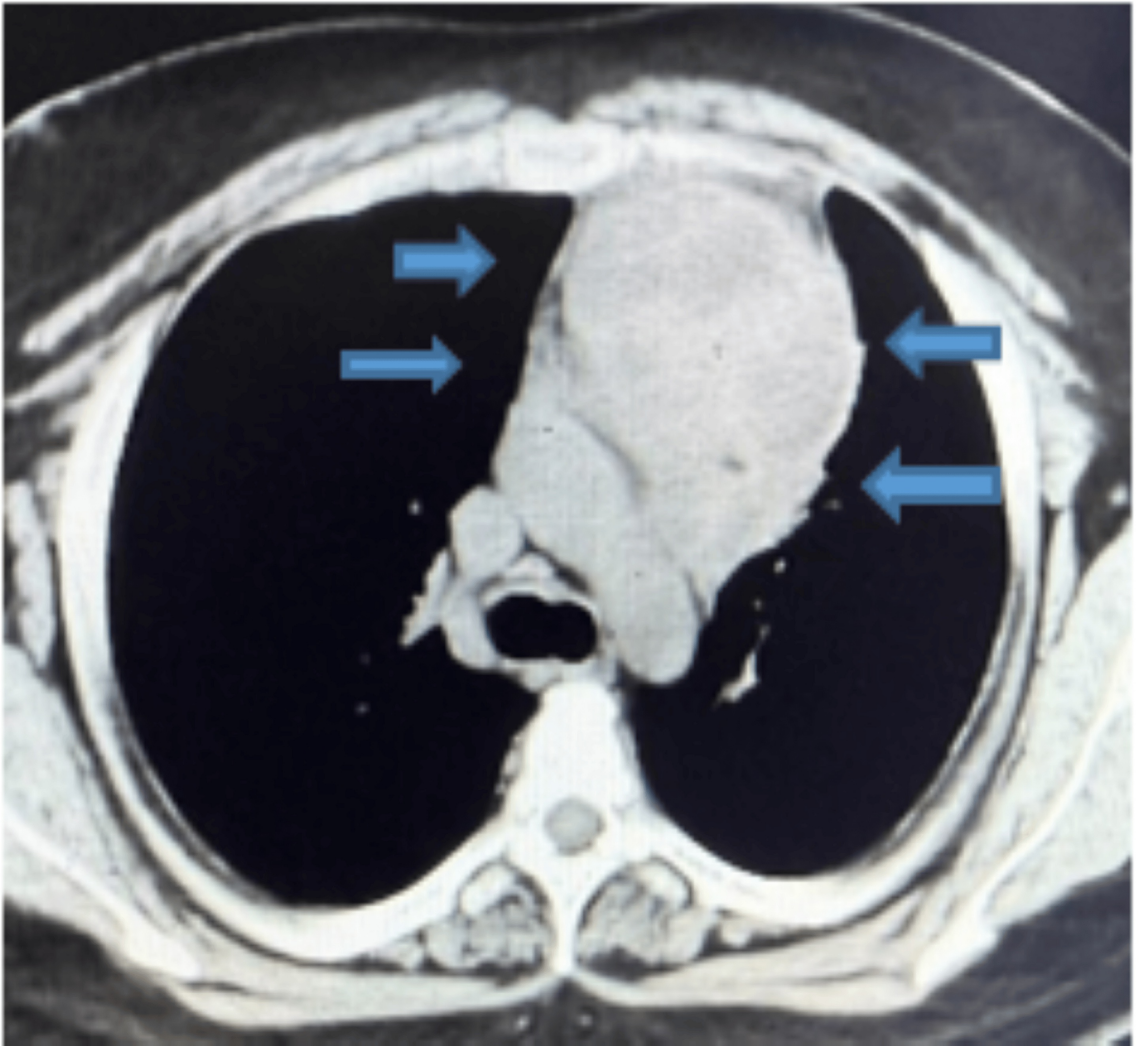
HRCT chest at presentation showing a large necrotic anterior mediastinal mass HRCT, high-resolution computed tomography

A chest radiograph (Figure 1) at presentation demonstrated mediastinal widening, prompting cross-sectional imaging. A high-resolution chest CT was performed without intravenous contrast due to financial constraints. CT demonstrated a lobulated anterior mediastinal mass measuring 85 x 67 mm with central low attenuation areas consistent with necrosis. The lesion abutted the pericardium with focal thickening and showed loss of fat planes adjacent to the aortic arch. A subcarinal lymph node measuring 27 x 20 mm was present. Mild fibronodular infiltrates were noted in the left upper lobe (Figure 2).

Laboratory evaluation revealed mild anemia (Hb 11.6 g/dL), elevated ESR (65 mm/hr), and mild transaminitis (AST 49 IU/L, ALT 59 IU/L). HIV testing was negative. Given the patient's age and prevascular mediastinal location, serum tumor markers were obtained: AFP: 3.59 ng/ml (reference range: 0.89-8.78); β-hCG: 1.50 mIU/ml (reference range: <5.00), all within normal limits (Table 1).

**Table 1:** Laboratory investigations with reference ranges on admission HB, hemoglobin; ESR, erythrocyte sedimentation rate; AST, aspartate aminotransferase; ALT, alanine aminotransferase; AFP, alpha-fetoprotein; β-hCG, beta-human chorionic gonadotropin

Test	Results	Units	Reference Range
HB	11.6	g/dl	13.0–17.0
ESR	18	mm/hr	0–10
Bilirubin total, serum	1.4	mg/dl	0.2–1.2
Bilirubin direct, serum	0.5	mg/dl	< 0.3
Bilirubin Indirect, Serum	0.9	mg/dl	-
AST, serum	49	U/L	5–34
ALT, serum	59	U/L	0–41
AFP	3.59	ng/ml	0.89–8.78
β-hCG	1.50	mIU/ml	< 5.00

**Figure 3:**
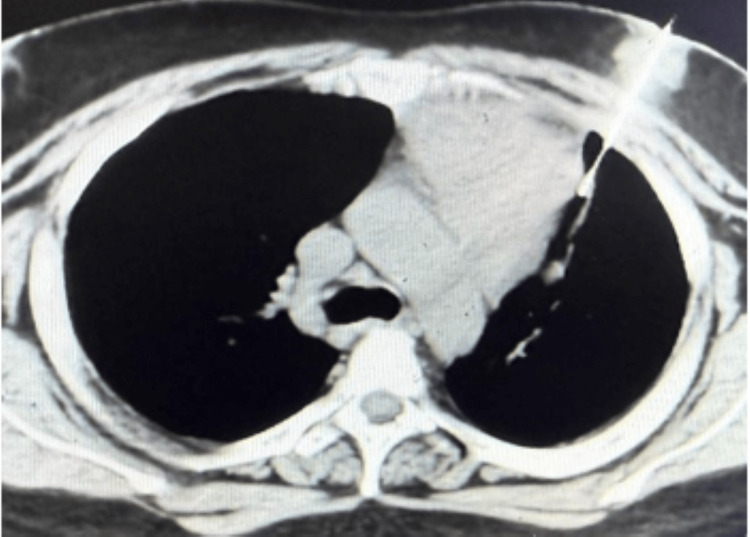
CT-guided biopsy

**Figure 4:**
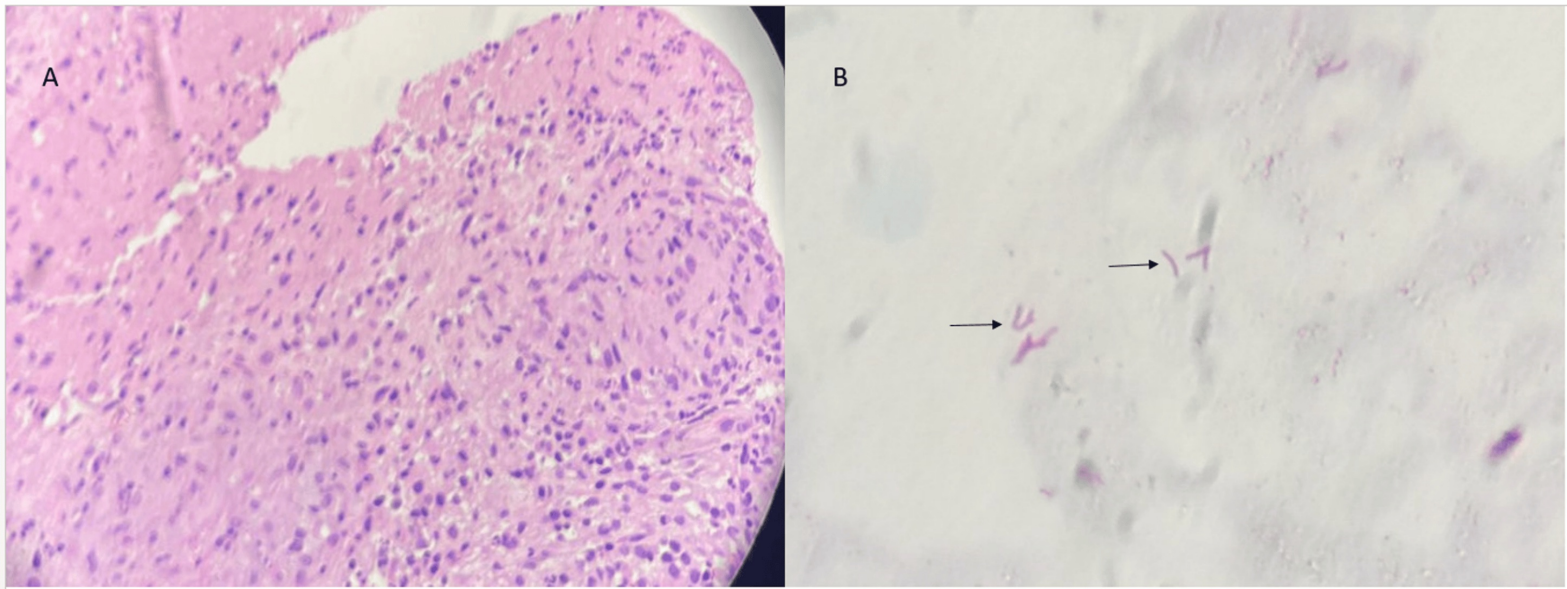
Histopathological findings: (A) hematoxylin and eosin-stained (40×) section showing well-formed caseating epithelioid cell granulomas with Langhans-type multinucleated giant cells in the background of necrosis. (B) Ziehl-Neelsen-stained (100×) section demonstrating AFB (arrow) AFB, acid-fast bacilli

The patient did not produce sputum, so sputum-based acid-fast bacilli (AFB) testing could not be performed. Because imaging characteristics could not reliably distinguish thymic tumor, lymphoma, and necrotizing infectious lymphadenitis, and because management would differ substantially among these possibilities, tissue diagnosis was prioritized. CT-guided core needle biopsy (Figure 3) demonstrated caseating granulomas with Langhans giant cells, and Ziehl-Neelsen staining identified AFB, confirming TB (Figure 4).

Treatment

Standard weight-based ATT was initiated (isoniazid, rifampicin, pyrazinamide, ethambutol). Liver function tests repeated after one week showed normalization (AST 30 IU/L, ALT 34IU/L). The patient was counseled regarding adherence and monitored clinically and biochemically during therapy.

Outcome and follow-up

**Figure 5:**
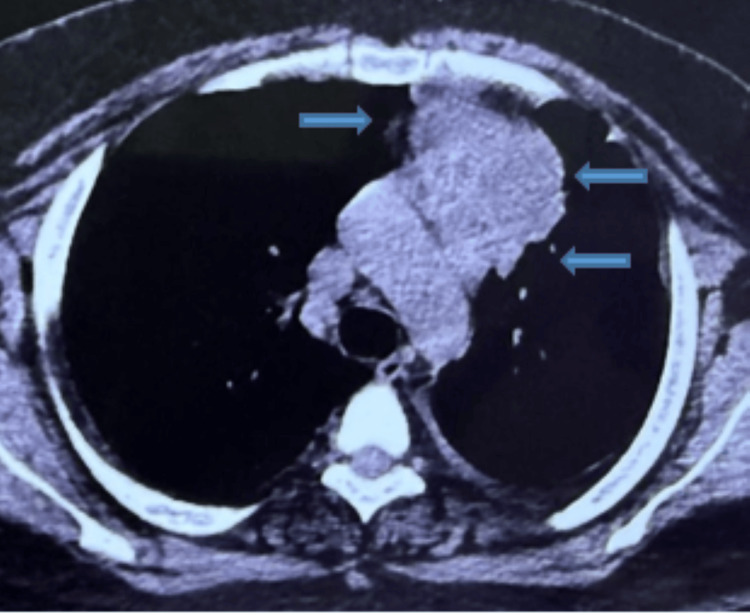
HRCT chest at two months demonstrating reduction in size and necrotic component of the mediastinal mass, confirming early radiologic response to ATT HRCT, high-resolution computed tomography; ATT, antitubercular therapy

**Figure 6:**
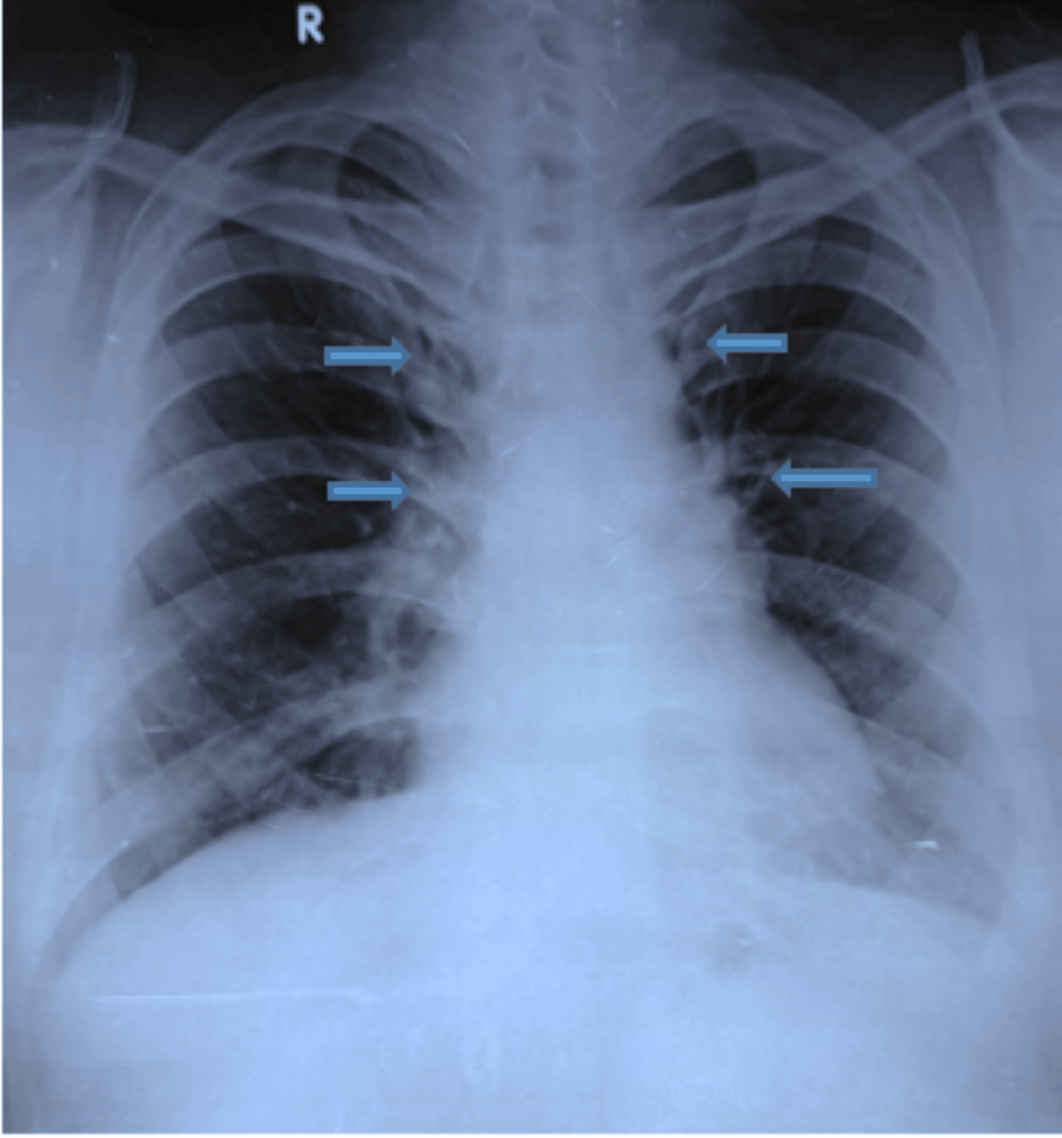
Chest radiograph (PA view) at six months demonstrating continued interval regression of mediastinal widening following therapy PA, posteroanterior

At two weeks, the patient reported complete resolution of fever and cough. Radiologic follow-up was documented sequentially, including baseline HRCT at admission, a repeat HRCT at two months showing partial regression of the anterior mediastinal mass to 70 x 62 x 35 mm (Figure 5), and chest radiographs at six months demonstrating further interval improvement (Figure 6).

**Figure 7:**
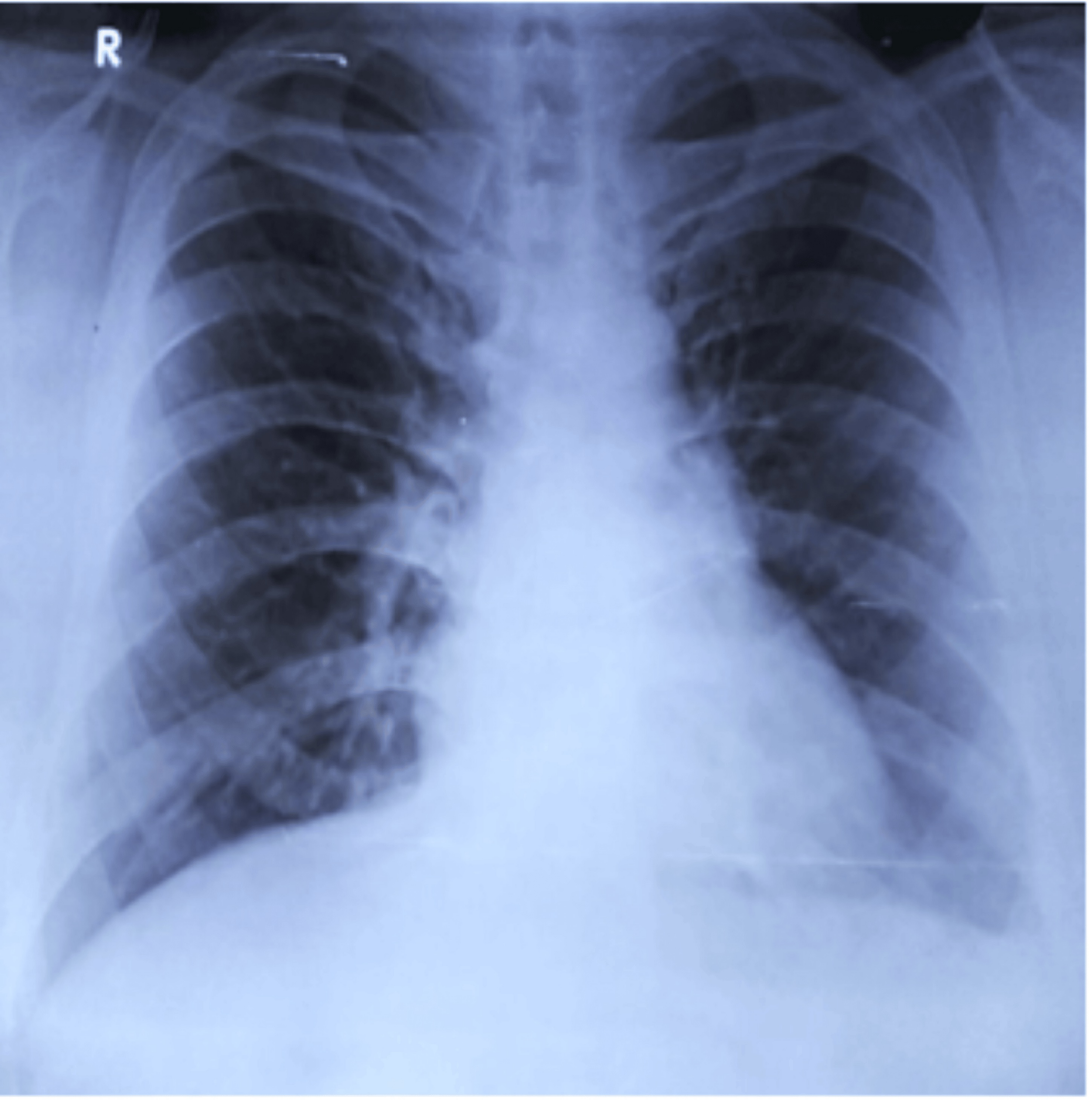
Chest radiograph (PA view) at eight months showing complete radiologic resolution, supporting cessation of ATT PA, posteroanterior view

Although six months is standard for extrapulmonary TB, given the initial large disease burden and extrapulmonary location, ATT was extended to a total duration of eight months, at which time chest radiography demonstrated complete radiologic resolution (Figure 7).

The patient remained asymptomatic throughout follow-up, and no clinical relapse was noted after radiologic resolution, after which ATT was discontinued.

## Discussion

This case highlights a key diagnostic pitfall in endemic settings: an anterior mediastinal TB mass in an immunocompetent young adult can closely mimic the classic anterior mediastinal differential and appear radiologically malignant. A compartment-based framework remains central to evaluating mediastinal lesions, and the ITMIG classification provides a standardized CT-based approach that helps localize disease, narrow differentials, and guide procedural planning [1]. Radiologically, TB-related mediastinal masses often demonstrate central necrosis, conglomerate lymphadenopathy, and preservation of surrounding structures, whereas malignant lesions may show invasive behavior; however, substantial overlap exists, limiting reliable distinction based on imaging alone. In our patient, constitutional symptoms (fever and weight loss), mediastinal widening on chest radiography, and a large lobulated necrotic prevascular mass with pericardial abutment strongly raised concern for lymphoma or thymic malignancy at presentation. Germ cell tumor was also considered, given the patient's age and lesion location; however, AFP and β-hCG were within normal limits, and there was no palpable gonadal/inguinal mass on examination, reducing support for a malignant mediastinal germ cell tumor in this clinical context. Even with these markers, imaging alone could not reliably differentiate lymphoma, thymic pathology, and necrotizing infectious lymphadenitis, and definitive management would have diverged substantially among these entities, making timely tissue confirmation essential.

Case literature reinforces that isolated anterior mediastinal TB in immunocompetent patients is uncommon and often diagnosed only after histologic confirmation because clinical and radiologic overlap with malignancy is significant [2]. Maguire et al. described an immunocompetent young man in whom bronchoscopy with lavage and mucosal biopsies were nondiagnostic, and mediastinoscopy was ultimately required to establish TB by demonstrating necrotizing granulomatous inflammation with AFB [2]. Maeder et al. similarly illustrate that necrosis can pose a sampling challenge: CT-guided aspiration yielded only necrotic material and was nondiagnostic, necessitating mediastinoscopy for definitive diagnosis; their report also documented a gradual radiologic response over months [3]. In contrast, our patient’s diagnosis was established on the first attempt with CT-guided core biopsy, demonstrating caseating granulomas with Ziehl-Neelsen-positive AFB, enabling prompt initiation of ATT with rapid symptomatic improvement.

Beyond diagnostic confirmation, our case adds practical educational value by documenting a complete radiologic trajectory. Khilnani et al. reported a large anterior mediastinal TB mass (7.5 × 6.0 cm) causing tracheal compression, diagnosed by CT-guided biopsy, with improvement evident over months and near-complete resolution reported by approximately nine months, supporting the concept that radiologic regression often lags behind early clinical recovery [4]. Our patient similarly showed early symptomatic resolution within two weeks, objective reduction in mass dimensions on HRCT at two months, further interval improvement on chest radiography at six months, and complete radiographic resolution by eight months, allowing discontinuation of therapy. Taken together, these cases support maintaining TB high in the differential diagnosis of necrotic anterior mediastinal masses in endemic regions, even among immunocompetent patients, and emphasize that early acquisition of adequate tissue can prevent unnecessary oncologic therapy or invasive surgical intervention.

Finally, the diagnostic pathway in our patient reflects a pragmatic, resource-limited setting: contrast-enhanced CT was not performed due to financial constraints, yet a definitive diagnosis was still achieved through image-guided core biopsy. CT-guided core sampling is an established approach for anterior mediastinal masses, with a favorable diagnostic yield in published series [5] and supportive overall performance and safety in systematic review/meta-analysis data for mediastinal masses [6]. This reinforces a practical message for clinicians: when an anterior mediastinal mass is necrotic, and malignancy is suspected, tissue diagnosis, preferably with core sampling when feasible, remains the pivotal step to secure a diagnosis and direct curative therapy [1,5,6].

A brief comparison of our case with previously reported immunocompetent anterior mediastinal TB cases is provided in Table 2 [2-4].

**Table 2:** Comparison of immunocompetent anterior mediastinal TB cases with the present case M, male; CXR, chest X-ray; CT, computed tomography; HRCT, high-resolution computed tomography; CECT, contrast-enhanced computed tomography; AFB: acid-fast bacilli; SVC, superior vena cava

Feature	Present Case	Khilnani et al., 2011 [4]	Maguire et al., 2016 [2]	Maeder et al., 2003 [3]
Age/sex	Late 20s/M	14/M	22/M	22/M
Symptoms	Dry cough, low-grade fever, 8 kg weight loss	Fever (nine months), weight loss (8 kg), mild dry cough	Chest pain, positional dyspnea, dry cough, dysphagia	Fever, night sweats, weight loss, dyspnea, palpitations
Initial CXR	Mediastinal widening	Mediastinal mass	Right paratracheal stripe enlargement/anterior mass	Widened upper mediastinum
CT modality	HRCT non-contrast	CECT described	CECT described	CECT described
Mass size	85 × 67 mm → 70 × 62 × 35 mm at 2 months	7.5 × 6.0 cm	Large (dimensions not stated)	Large (dimensions not stated)
Key feature	Pericardial abutment; aortic arch fat plane loss	Tracheal compression	Multistation mediastinal nodes; relations to SVC/pulmonary vessels	Right atrial infiltration; great vessel involvement; SVC narrowing
Diagnostic path	CT-guided core biopsy diagnostic	CT-guided biopsy diagnostic	Bronchoscopy nondiagnostic → mediastinoscopy	CT-guided aspiration nondiagnostic → mediastinoscopy
AFB result	Positive on tissue stain	Negative on stain (reported)	Positive on tissue stain	Positive on tissue stain
Outcome	Complete resolution by eight months	Near-complete resolution by 12 months	Interval improvement on follow-up CXR, treatment completed at six months	Near-complete resolution by nine months

## Conclusions

TB should be consistently considered in the differential diagnosis of necrotic anterior mediastinal masses even in immunocompetent young adults, particularly in regions where TB remains endemic. Radiologic appearances in this setting may closely resemble malignant conditions such as thymic tumors or lymphoma, and reliance on imaging alone can lead to diagnostic delay or inappropriate management. When clinical and radiologic features overlap with malignancy and therapeutic pathways differ substantially, early histopathological confirmation becomes crucial. Image-guided core needle biopsy, when technically feasible, offers a reliable and minimally invasive means of establishing a definitive diagnosis. Prompt recognition and initiation of ATT can result in rapid clinical improvement and complete radiologic resolution while avoiding unnecessary surgical intervention in this curable disease entity.
